# Pseudoaneurysm after total arch replacement mimicking malignant lymphadenopathy

**DOI:** 10.1002/rcr2.645

**Published:** 2020-08-19

**Authors:** Yosuke Chiba, Kei Yamasaki, Hiroaki Ikegami, Kazuhiro Yatera

**Affiliations:** ^1^ Department of Respiratory Medicine University of Occupational and Environmental Health, Japan Kitakyushu Japan

**Keywords:** Lymphadenopathy, pseudoaneurysm, total arch replacement

## Abstract

Pseudoaneurysm should be considered in the differential diagnosis when the computed tomography (CT) findings show a mediastinal mass in patients with a history of cardiovascular surgery even if such surgery occurred over two decades previously.

## Graphical abstract

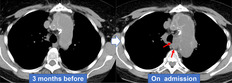

## Clinical Image

**Figure 1:**
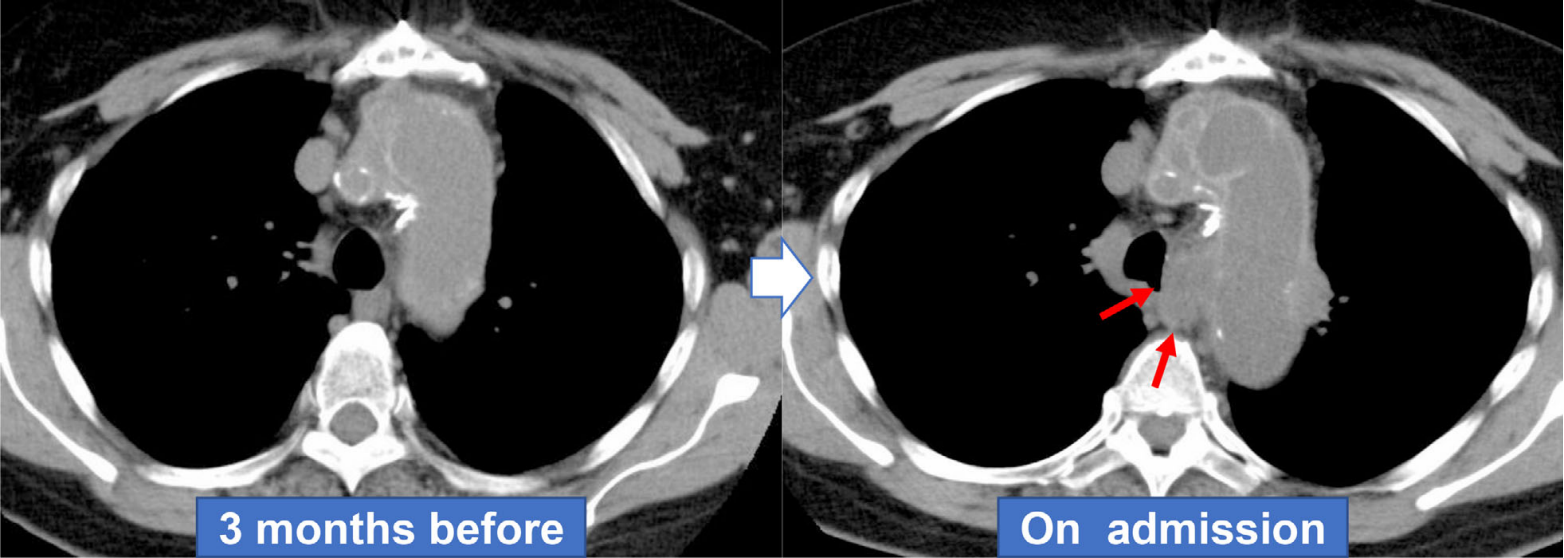
An abnormal mediastinal mass‐like lesion is observed in the para‐tracheal region on non‐contrast computed tomography (NCCT).

**Figure 2:**
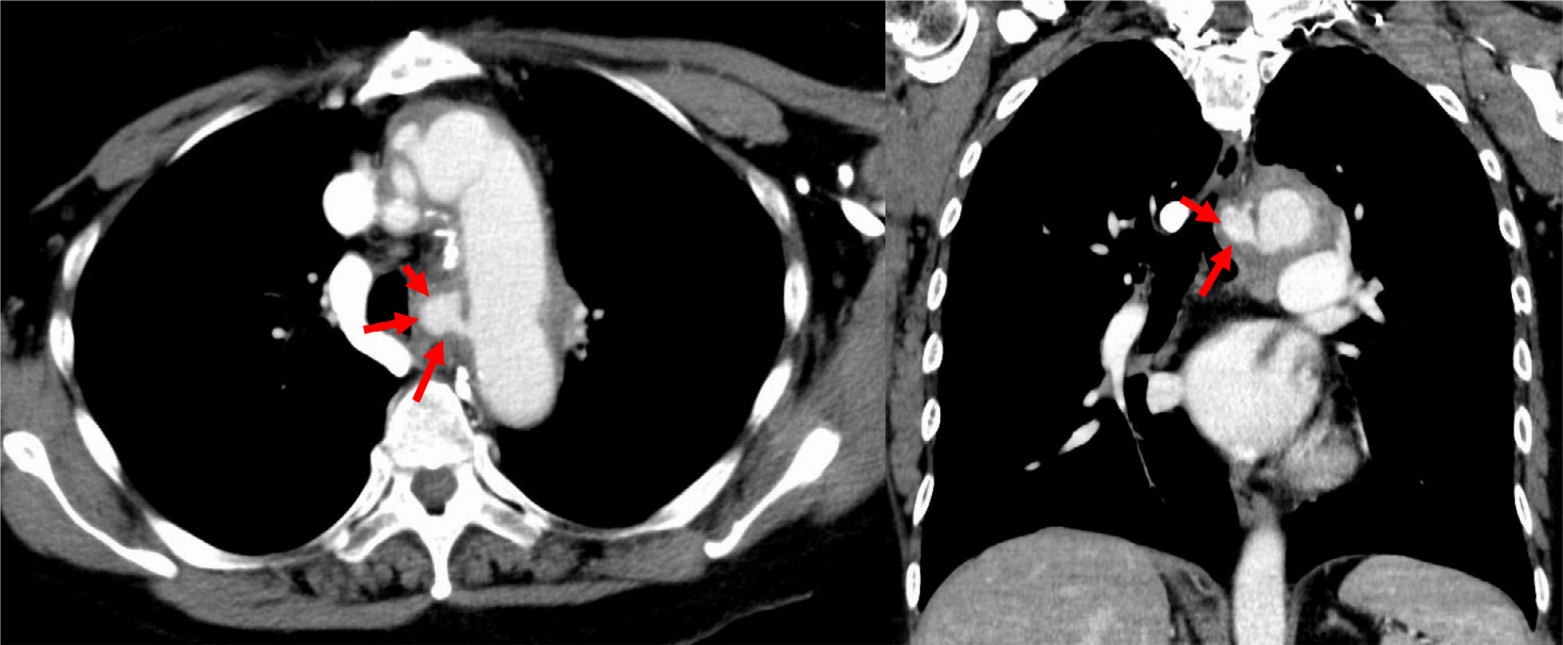
Contrast medium extravasation from the artificial blood vessel of the aortic arch on chest tomography angiography (6 sec after the contrast agent bolus injection).

A 68‐year‐old Japanese woman with a surgical history of total aortic arch replacement for a distal aortic arch aneurysm 20 years earlier visited our hospital because of back pain for three days and haemoptysis. Chest non‐contrast computed tomography (NCCT) was performed because of a contrast agent allergy and showed an abnormal mediastinal mass‐like lesion in the subaortic region (Fig. 1). The radiologist and pulmonologist were unable to definitively diagnose the mediastinal mass as a neoplastic or vascular abnormality, so computed tomography (CT) angiography was performed after pre‐medication, which revealed contrast medium extravasation from the vicinity of the anastomotic site of the artificial blood vessel of the aortic arch, suggestive of pseudoaneurysm in the mediastinal area (Fig. 2). She was immediately transferred to the cardiovascular surgery department and underwent emergency stent graft insertion. A pseudoaneurysm occurring a long time after thoracic replacement is extremely rare, and only one case has been previously reported in which the patient developed a pseudoaneurysm more than 20 years after undergoing cardiac surgery [1]. In this patient, CT angiography should have been performed according to the chest CT protocol [2] because of her symptoms of haemoptysis and back pain. With the growing diversity in chest CT protocols, physicians need to choose the recommended protocols for individual indications.

### Disclosure Statement

Appropriate written informed consent was obtained for publication of this case report and accompanying images.
